# Is Contrast-Enhanced Ultrasound Superior to Computed Tomography for Differential Diagnosis of Gallbladder Polyps? A Cross-Sectional Study

**DOI:** 10.3389/fonc.2021.657223

**Published:** 2021-05-24

**Authors:** Zhiqing Yuan, Xuesong Liu, Qiwei Li, Yunhe Zhang, Ling Zhao, Fenghua Li, Tao Chen

**Affiliations:** ^1^ Department of General Surgery, Renji Hospital, School of Medicine, Shanghai Jiao Tong University, Shanghai, China; ^2^ Department of Ultrasound, RenJi Hospital, School of Medicine, Shanghai Jiao Tong University, Shanghai, China; ^3^ Department of Critical Care Medicine, Shanghai East Hospital, School of Medicine, Tongji University, Shanghai, China; ^4^ Department of Pathology, Renji Hospital, School of Medicine, Shanghai Jiao Tong University, Shanghai, China; ^5^ Department of Biliary-Pancreatic Surgery, Renji Hospital, School of Medicine, Shanghai Jiao Tong University, Shanghai, China

**Keywords:** gallbladder polypoid lesions, non-neoplastic polyp, neoplastic polyp, computed tomography, contrast-enhanced ultrasonography

## Abstract

**Objective:**

To compare the clinical value of contrast-enhanced ultrasonography (CEUS) versus computed tomography (CT) for distinguishing neoplastic and non-neoplastic gallbladder polyps. Given whether laparoscopic cholecystectomy is needed, differential diagnosis of neoplastic and non-neoplastic gallbladder polyps is more important than benign and malignant polyps.

**Methods:**

A total of 89 consecutive patients with polypoid lesions of the gallbladder > 10 mm in size without local invasion or distant metastasis during primary screening were enrolled in this prospective and comparative study. All patients who met the inclusion criteria underwent CEUS and CT examinations prior to surgical resection. The enhancement patterns and microvascular imaging types were analyzed on CEUS. The maximum diameter and CT value of the lesions were also recorded and subjected to a comparative analysis. The clinical value of the two diagnostic methods is compared.

**Results:**

Of the 89 patients, there were 58 (65.2%) cases of non-neoplastic polyps and 31 (34.8%) cases of neoplastic polyps. The average diameter of neoplastic polyps was significantly higher than that of non-neoplastic polyps (P<0.001). The detection rate using CEUS was 100%. The proportion of perceived non-neoplastic polyps in the nonenhanced and arterial phases were 48.3% and 77.6%, respectively, which were significantly lower than those of neoplastic polyps (93.5%, P<0.001 and 100.0%, P<0.001, respectively). However, in the venous and delayed phases, all cholesterol polyps and neoplastic polyps were perceived. CT showed that non-neoplastic polyps exhibited delayed enhancement. On CEUS 29.0% neoplastic polyps showed a perfusion defect, whereas 6.9% non-neoplastic polyps showed a perfusion defect (P=0.005). The microvascular architecture of the lesions on CEUS was categorized into 4 types: spotty, linear, branched, and spinous, and there were significant differences between the two groups (P<0.001). The sensitivities and specificities were 87.10% and 68.97% for CEUS and 83.87% and 77.59% for CT, respectively (P=0.406).

**Conclusions:**

CEUS and CT are useful for differential diagnosis of neoplastic and nonneoplastic polypoid lesions of the gallbladder. Diagnostic efficacy was comparable between CEUS and CT. Thus, CEUS is preferred over CT in the differential diagnosis of neoplastic and non-neoplastic gallbladder polyps due to its comparable diagnostic efficacy and lack of radiation dose.

## Introduction

Gallbladder polyps are a relatively common disease with a reported prevalence of 4–7% (1, 2). In recent years, the widespread application of abdominal ultrasonography has led to an increase in the detection of gallbladder polyps (3). Gallbladder polyps can be categorized into two types: neoplastic and non-neoplastic. Neoplastic polyps include adenomas, adenocarcinomas, squamous cell carcinomas, and other malignancies. These polyps are either malignant or have a malignant tendency, and surgery is necessary to obtain a good prognosis. Non-neoplastic polyps mainly include cholesterol polyps, inflammatory polyps, adenomyosis, and fibromas. Regular follow-up assessments are sufficient for non-neoplastic polyps (4, 5). Given the distinct treatment strategies, differential diagnosis of neoplastic and non-neoplastic gallbladder polyps is more important than benign and malignant polyps

Multiple imaging methods, including ultrasound, contrast-enhanced ultrasonography (CEUS), computed tomography (CT), endoscopic ultrasonography (EUS), magnetic resonance imaging (MRI), and even positron emission tomography (PET), have been used for the differential diagnosis of gallbladder polyp types. In view of increased patient comfort and lower costs, EUS, MRI, and PET are less common than the other methods. At Renji hospital in Shanghai, CT is widely used to detect and differentiate gallbladder polyps. However, due to the low detection rate, CT is not strongly recommended for gallbladder polyps. Some studies have suggested that CT is less effective than traditional ultrasound for diagnosing gallbladder disease (6, 7). Recent technical developments, such as high-resolution computed tomography (HRCT), have greatly improved the image resolution of CT. During CT imaging parameters are selected to maximize the spatial resolution, which may enhance the diagnostic capacity of this method.

CEUS visualizes the vascular patterns of gallbladder polyps and provides real-time images. CEUS should be performed prior to conventional ultrasonography (8, 9). However, as a new method, many clinicians are unaccustomed to using CEUS. To the best of our knowledge, the comparison of the diagnostic value of CEUS and CT for gallbladder polyps is unknown. Thus, the preferred examination method for clinical assessment of gallbladder polyps remains unclear. In the present study, we aimed to investigate the diagnostic performances of CEUS and CT for the preoperative differential diagnosis of gallbladder polyps and to compare the diagnostic accuracies of these modalities.

## Materials and Methods

### Patients

From August 2016 to October 2019, 420 patients with gallbladder polypoid lesions diagnosed by conventional transabdominal ultrasonography were prospectively enrolled in this study. This study was approved by the Ethics Committee of Renji Hospital and was registered at ClinicalTrials.gov (NCT02762227). Written informed consent was obtained from all patients enrolled in this study.

The patient exclusion criteria were as follows: (1) those with gallbladder polyps with a diameter < 10 mm; (2) those with lesions highly suspected to be cancerous due to local invasion or visible metastasis; (3) those with allergies to contrast agents or inability to undergo CT or CEUS; and (4) women who were pregnant or lactating. Ultimately, 89 patients with gallbladder lesions > 10 mm without evidence of local invasion or distant metastasis were included in this prospective comparative study. All patients underwent CT and CEUS before surgery.

### CT

CT examinations were performed using a 64-slice CT scanner (LightSpeed VCT, GE Healthcare, Milwaukee, WI, USA). Arterial phase scanning was triggered by the bolus tracking technique (+100 HU above the baseline) after injection of 100–120 mL nonionic contrast material (iopamidol 370 mg/mL; Bracco Sine Pharmaceutical Co., Ltd., Shanghai, China) into the antecubital vein at a rate of 3–4 mL/s. Portal venous and delayed phase scanning were performed with delays of 60 and 140 seconds after starting contrast medium injection, respectively. The CT parameter settings were as follows: 0.8 second tube rotation time, 120 kVp, 140–250 mAs, 0.625 mm collimation, 5.0 mm slice thickness for axial images, pitch of 1.375, 1.25 mm reconstruction slice thickness, 1.25 mm reconstruction interval, and standard reconstruction algorithm. Iopamidol (Haibo Pharmaceutical Company, Shanghai, China) was used as the contrast agent. Its main components were iopamidol and other ingredients including trometamol, disodium edetate, and hydrochloric acid. The specific concentration was 370 mg/mL. Image analysis was completed by two experienced radiology technicians with at least five years of experience.

### CEUS

CEUS examinations were performed using a Philips iU22 scanner (Philips Medical Solutions; Mountain View, CA, USA) with a 1-5 MHz vector transducer. Traditional transabdominal ultrasound and CEUS were performed by the same investigator with more than three years of experience in performing CEUS. Traditional ultrasound was initially used to locate the lesion. The bottom of the lesion was selected for further examination. When multiple lesions existed, the largest lesion was selected for evaluation. The target was placed in the center of the screen and kept stable. Pulse inversion harmonic imaging with an index of 0.06 was used. SonoVue (Bracco SpA, Milan, Italy), which contained sulfur hexafluoride microbubbles, was used as the contrast agent. The pulse repetition frequency was set at 7 Hz after injecting the contrast agent. First, 1.5 mL of contrast agent was injected within 2 seconds and the lesion was observed continuously for 5 minutes. Ten minutes after the first injection, 1.2 mL SonoVue was injected again for contrast enhanced microvascular imaging (MVI). When the lesion began to show enhancement, the MVI mode was initiated and the patient was instructed to hold their breath for at least 10 seconds. All subjects fasted for at least 8 hours prior to the examination. Image analysis was performed by two technicians with at least five years of experience who had assessed more than 150 cases of enhanced ultrasonography.

### Image Analysis

We used uniform diagnostic criteria for the differential diagnosis of gallbladder polyps. For CEUS images, cholesterol polyps were diagnosed if a mild enhancement pattern was observed on enhanced images and a spotty pattern was observed in MVI mode. Sharply pedunculated and well-defined margins were also taken into consideration. A marked enhancement pattern with a branched pattern in MVI mode and a well-defined margin were considered to indicate neoplastic polyps. Gallbladder cancer was considered when there was irregular mucosal or gallbladder wall thickening with or without the loss of normal mural layers. The lesion was heterogeneous and blood flow appeared spinous in MVI mode of gallbladder cancer.

According to previously published literature and our experience (10), the enhancement patterns on CEUS were classified as follows: (1) enhancement homogeneity, defined as homogeneous enhancement when the lesion was enhanced at the same extent throughout or heterogeneous enhancement when the lesion was enhanced at different extents in different locations; (2) enhancement degree, defined as marked enhancement when the enhancement extent was greater than or equal to that of adjacent liver tissue at the time of peak intensity, mild enhancement when the enhancement extent during the whole contrast enhanced sonographic process was less than that of liver tissue, or none when no enhancement was shown; (3) presence of a perfusion defect; and (4) distribution of intralesional vascularity on microvascular imaging, which was categorized into branched, linear, spotty, and spinous vessel types.

CT image analysis was completed by two senior radiologists who were blinded to the patient’s pathologic diagnosis. When their evaluations were inconsistent, the final evaluation was performed by a chief radiologist. The perception of polyps in the different dynamic phases was performed first, followed by measurement of the size (maximum diameter) and CT value (assessed in HUs) of the polyps. Oval-shaped regions of interest (ROIs) for the whole gallbladder were manually placed at the perception scan phase containing the maximum validation area of ​​the polyp. The ROIs were automatically duplicated to the material decomposition images in exactly the same position and slice in which they were manually placed. The maximum diameter of the polyp was measured in the perception scan phase with the maximum validation area of ​​the polyp. The CT values ​​of the polyp was measured within the ROIs in the plain phase, arterial phase, portal venous phase, and delayed phase. For multiple polyps, the largest polyp was used as the measurement target. Similar diagnostic criteria were applied for CT images. Polyp size, shape (sessile or pedunculated), defined margins, and visual appearance were all taken into account. Based on the technicians’ experience, polyps that could not be displayed in either the nonenhanced phase or arterial phase tended to be classified as non-neoplastic polyps. If the polyp could not be detected in any phase, we directly classified it as a non-neoplastic polyp.

### Surgery

All patients underwent cholecystectomy (laparoscopic or open) after imaging examination. The resected specimens were immediately fixed in 10% buffered formalin and embedded in paraffin. Sections then underwent routine hematoxylin-eosin staining.

### Data Collection and Statistical Analysis

All imaging data were collected prospectively and analyzed before surgery. Each technician made the diagnosis independently. In cases where the two technicians reached different diagnoses, a third technician was consulted. Clinical diagnoses were compared with histopathologic results to assess the diagnostic accuracy of each modality. Data were compared between groups using the χ^2^ test and t-test. P values <0.05 were considered to be statistically significant. All statistical analyses were performed using SPSS software version 19.0.

## Results

### Patient Characteristics

**Figure 1:**
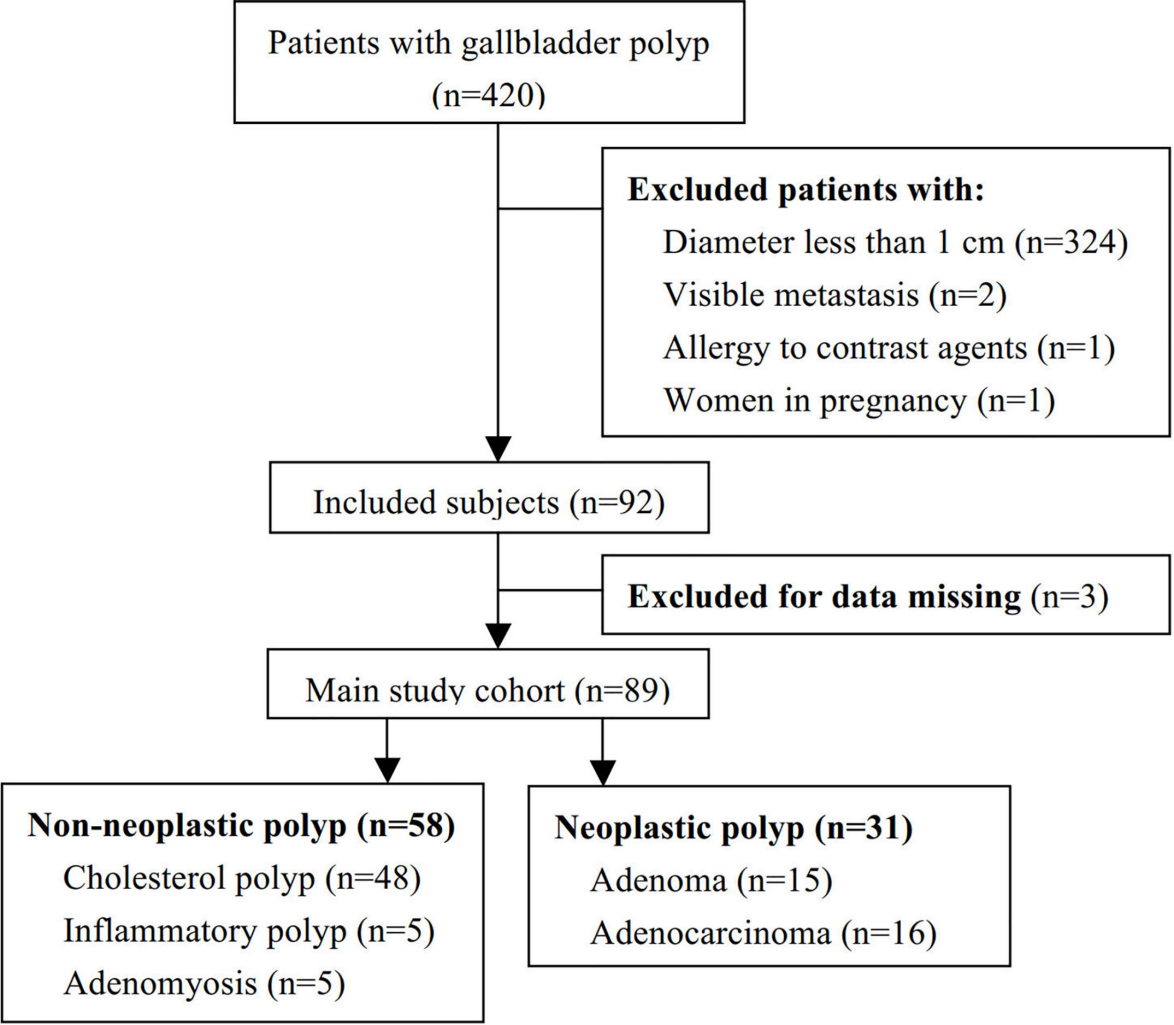
Flow chart of the patient inclusion process.

A total of 420 patients with gallbladder polypoid lesions visited our center, of which 92 met the inclusion criteria. Three patients were excluded due to a rare pathological type: one was melanoma and two were xanthogranuloma. Thus, 89 patients were included for further analysis (**Figure 1**). The mean patient age was 53.96 years (range: 23–89). Twelve (13.5%) patients had gallbladder stones as well as polyps. There were 58 (65.2%) cases of non-neoplastic polyps, including 48 cases of cholesterol polyps, 5 cases of inflammatory polyps, and 5 cases of adenomyosis. There were 31 (34.8%) cases of neoplastic polyps, including 15 adenomas and 16 adenocarcinomas. Cholesterol polyps were the most common type, followed by neoplastic polyps. A significant difference was observed in age between the non-neoplastic and neoplastic polyp groups. The general patient characteristics are reported in **Table 1**.

**Table 1:** Patient characteristics.

Characteristics	Non- neoplastic polyps	Neoplastic polyps	P
	n=58	n=31	
Age (year)	51.50 ± 14.63	58.55 ± 14.03	0.031
Sex (male/female)	23/35	15/16	0.428
Associated GB stone	7	5	0.593
BMI (kg/m^2^)	24.23 ± 3.41	23.92 ± 3.35	0.683
Hypertension	8	5	0.766
Diabetes	7	3	0.516
Hypercholesterolemia	7	2	0.639
Smoking	4	3	0.959
Drinking	2	3	0.464

### Difference in Detection Rate Between CT and CEUS

**Figure 2:**
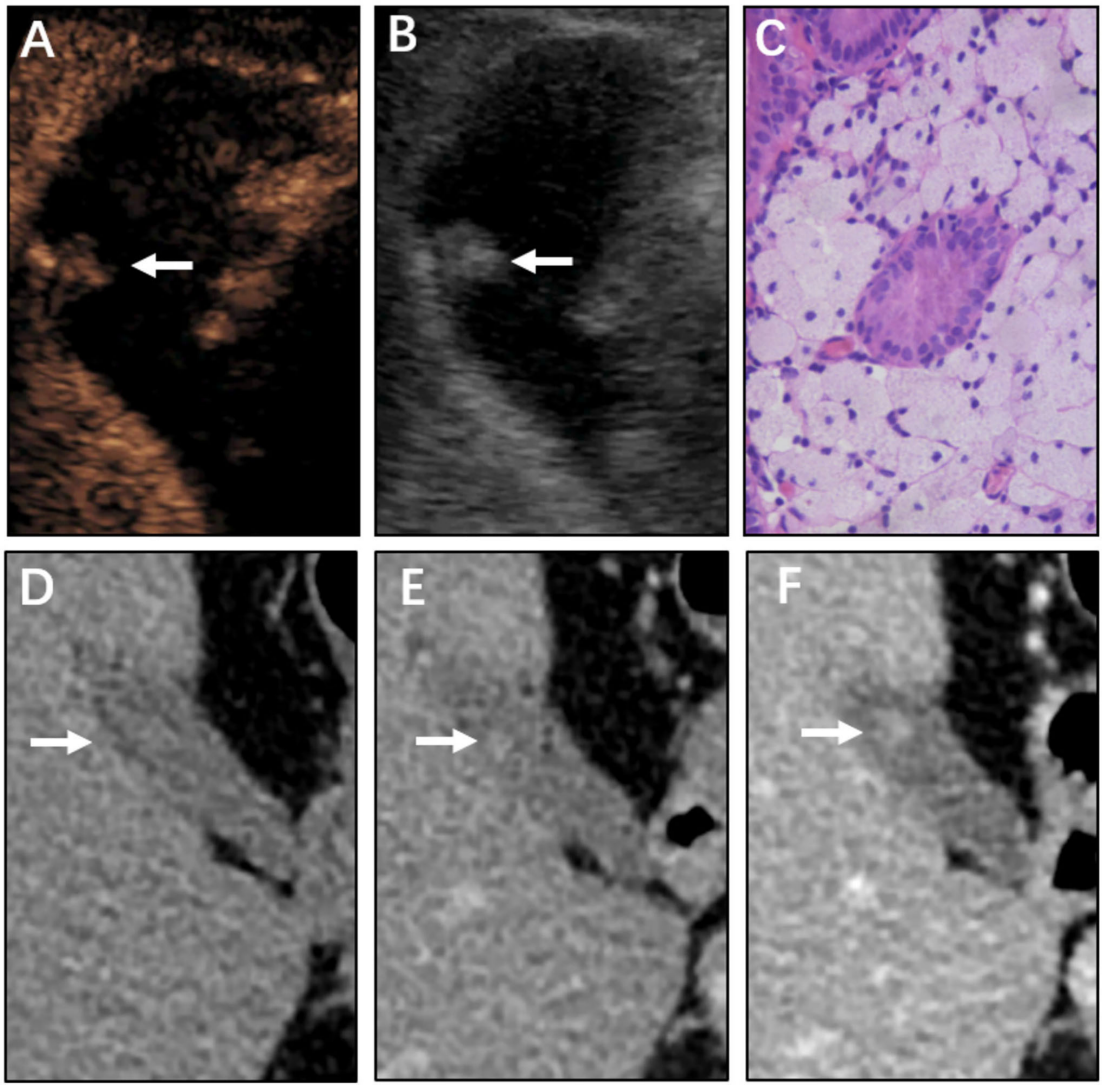
Non-neoplastic polyp (cholesterol polyp) of the gallbladder in a 67-year-old man. CEUS image **(A, B)** showing an 11 mm sized mass. **(A)** Microvascular image showing intralesional vessels with a spotty pattern. **(B)** 2D mode. **(C)** Histopathologic image of a gallbladder cholesterol polyp. (H&E stained, original magnification × 400). CT images **(D–F)** show the same mass in the nonenhanced phase, arterial phase, and portal venous phase, respectively. The mass could barely be detected in the nonenhanced phase. As the contrast agent was perfused, the shape of the mass gradually became clear.

**Figure 3:**
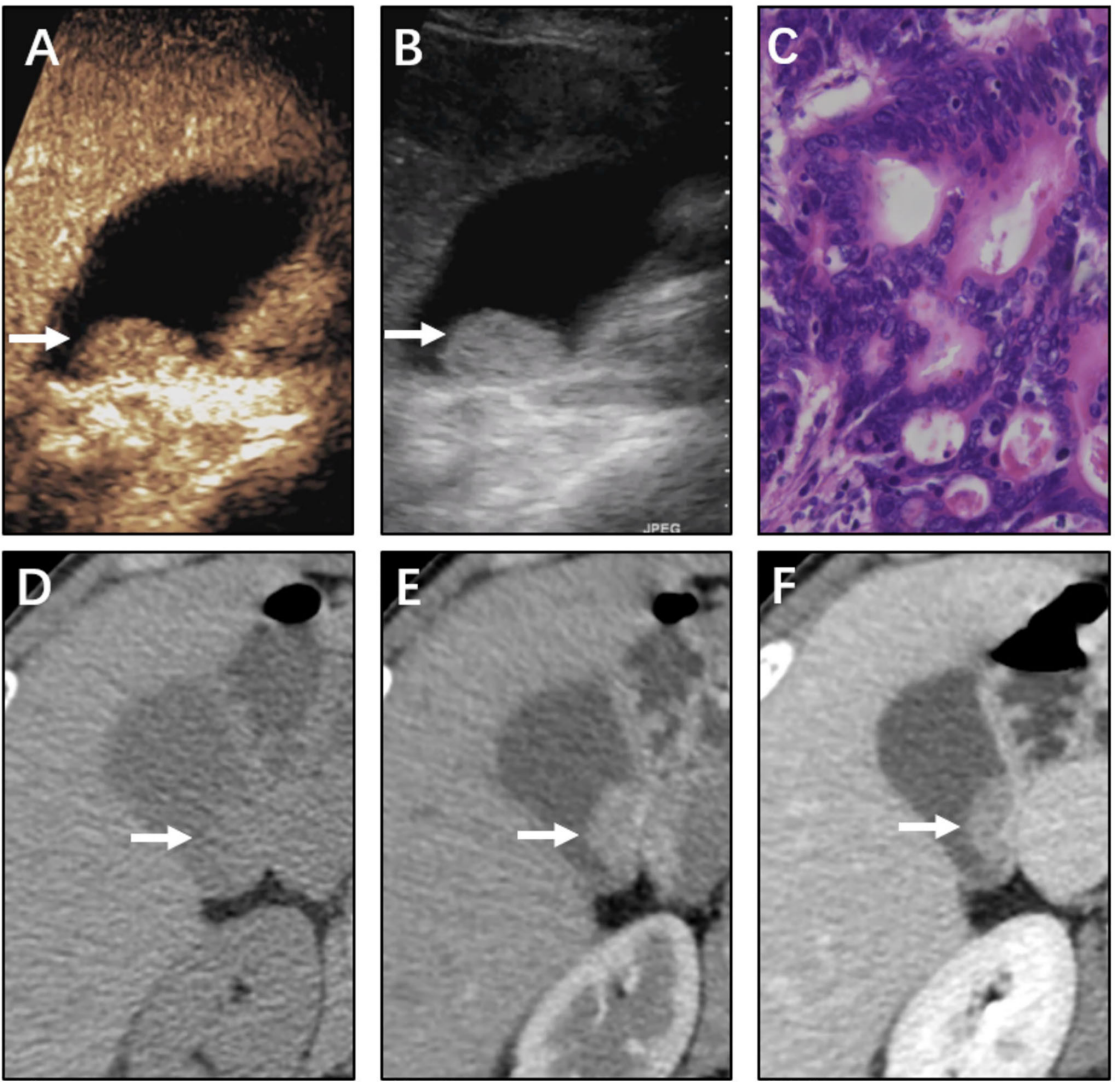
Neoplastic polyp (adenocarcinoma) of the gallbladder in a 61-year-old woman. CEUS image **(A, B)** showing a 21 mm sized mass. **(A)** Microvascular image showing intralesional vessels with a strong and heterogeneous pattern. **(B)** 2D mode. **(C)** Histopathologic image of gallbladder adenocarcinoma. (H&E stained, original magnification × 400). CT images **(D–F)** showing the same mass in the nonenhanced phase, arterial phase, and portal venous phase, respectively. The mass was clearly detected in the nonenhanced phase. The mass was significantly enhanced with the use of a contrast agent.

**Figure 4:**
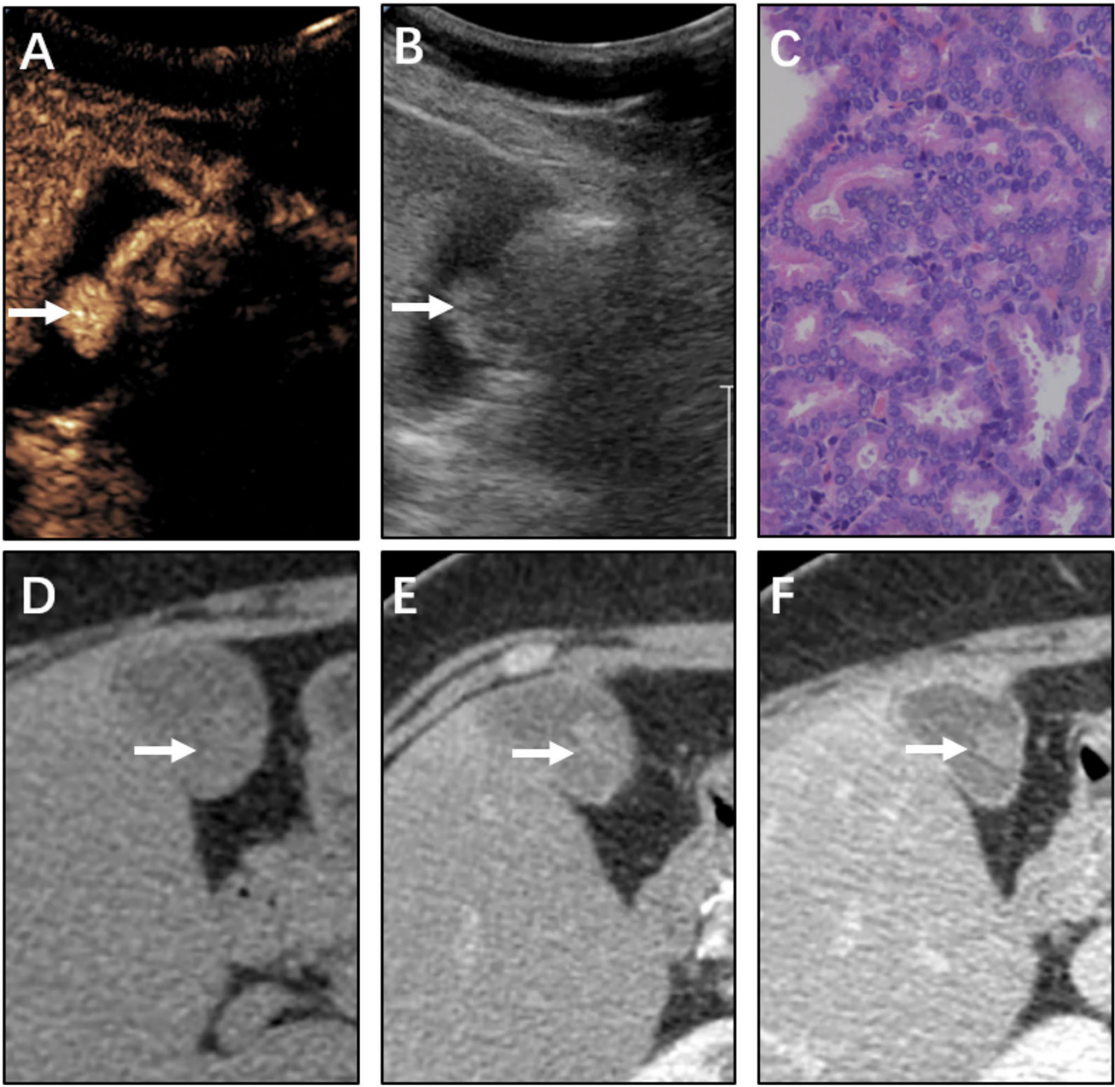
Neoplastic polyp (adenoma) of the gallbladder in a 61-year-old man. CEUS image **(A, B)** showing a 16 mm sized mass. **(A)** Microvascular image showing intralesional vessels with a branched pattern compared with **(B)** in 2D mode. **(C)** Histopathologic image of gallbladder adenoma. (H&E stained, original magnification × 400). CT images **(D–F)** showing the same mass in the nonenhanced phase, arterial phase, and portal venous phase, respectively. The mass could be vaguely detected in the nonenhanced phase. The mass was gradually enhanced as the contrast agent was perfused. The CT value peak of the mass was lower than that of adenocarcinoma in the arterial phase and portal venous phase.

Compared with the 100% detection rate using CEUS,CT could not visualize all lesions in nonenhanced phase. For non-neoplastic and neoplastic polyps in nonenhanced phase, the detection rates were 48.3% (28/58) and 93.5% (29/31), respectively (P<0.001). In arterial phase, the detection rate of neoplastic polyps was significantly higher than non-neoplastic polyps (77.6% vs 100%, P=0.004). All cases were detected in the portal venous phase (**Table 2**, **Figures 2**–**4**). Dynamic analysis of the perception of polyps in consecutive phases of contrast-enhanced CT scans showed that non-neoplastic polyps exhibited delayed enhancement.

**Table 2:** Detection rate of CT in the non-enhanced phase and enhanced phase.

Pathology results	Non-enhanced Phase (displayed/total)	Arterial Phase (displayed/total)	Portal venous phase (displayed/total)
Non-neoplastic	28/58 (48.3)	45/58 (77.6)	58/58 (100.0)
Cholesterol polyp	19/48 (39.6)	35/48 (72.9)	48/48 (100.0)
Inflammatory polyp	4/5 (80.0)	5/5 (100.0)	5/5 (100.0)
Adenomyosis	5/5 (100.0)	5/5 (100.0)	5/5 (100.0)
Neoplastic	29/31 (93.5)	31/31 (100.0)	31/31 (100.0)
Adenoma	13/15 (86.7)	15/15 (100.0)	15/15 (100.0)
Adenocarcinoma	16/16 (100.0)	16/16 (100.0)	16/16 (100.0)
P	<0.001	0.004	/

Data are presented as number (percent).

The average diameter of neoplastic polyps was 21.36 ± 7.45 mm, which was significantly higher than that of non-neoplastic polyps (10.28 ± 2.90 mm, P<0.001, **Table 3**). To quantitatively assess delayed enhancement in non-neoplastic polyps, the CT values in all phases were measured and analyzed. The CT values in two groups increased after contrast agents were intravenously injected and filled the polyps. The CT values of neoplastic polyps in the nonenhanced phase and arterial phase were significantly high than those of non-neoplastic polyps (both P<0.001, **Table 3**), whereas no significant differences were observed in the portal venous phase or delayed phase.

**Table 3:** CT parameters of neoplastic and non-neoplastic polypoid lesions.

	Non- neoplastic polyps	Neoplastic polyps	P
	n=58	n=31	
Diameter (mm)	10.28 ± 2.90	21.36 ± 7.45	<0.001
Hounsfield unit			
Nonenhanced phase	22.35 ± 7.88	38.22 ± 8.44	<0.001
Arterial phase	40.84 ± 18.22	60.48 ± 13.69	<0.001
Portal venous phase	75.98 ± 25.35	82.56 ± 17.27	0.578
Delayed phase	76.31 ± 15.43	73.36 ± 21.82	0.324

### CEUS Findings in Neoplastic and Non-Neoplastic Polyps

In terms of the enhancement intensity and pattern of CEUS, there was no significant difference between neoplastic polyps and non-neoplastic polyps (P=0.070 and 0.090, respectively, **Table 4**). Enhancement could not be as a sign of neoplastic lesion. 9 neoplastic polyps showed a perfusion defect on CEUS, whereas 4 non-neoplastic lesions showed a perfusion defect (29.0% vs 6.9%, P=0.005). The presence of a perfusion defect was helpful to distinguish between neoplastic and non-neoplastic polyps. On microvascular imaging, the microvascular architecture of 89 lesions was clearly delineated. Non-neoplastic lesions mainly showed the spotty pattern; neoplastic lesions tended to show the branched pattern. The microvascular architecture showed significant differences between neoplastic and non-neoplastic lesions (P<0.001, **Table 4**).

**Table 4:** CEUS features of neoplastic and non-neoplastic polypoid lesions.

Features	Non- neoplastic polyps	Neoplastic polyps	P
	n=58	n=31	
Enhancement intensity			0.070
Marked	43 (74.1)	28 (90.3)	
Mild	15 (25.9)	3 (9.7)	
None	0 (0)	0 (0)	
Enhancement pattern			0.090
Homogeneous	21 (36.2)	17 (54.8)	
Heterogeneous	37 (63.8)	14 (45.2)	
Perfusion defect			0.005
Present	4 (6.9)	9 (29.0)	
Absent	54 (93.1)	22 (71.0)	
Microvascular architecture			<0.001
Spotty	44 (75.9)	5 (16.1)	
Linear	8 (13.8)	7 (22.6)	
Branched	5 (8.6)	12 (38.7)	
Spinous	1 (1.7)	7 (22.6)	

Data are presented as number (percent).

### Comparison of Diagnostic Accuracy of CT and CEUS

In terms of diagnostic accuracy, CT and CEUS performed comparably at differentiating between neoplastic and non-neoplastic gallbladder polyps. The overall sensitivity of CT was 83.87% and that of CEUS was 87.10%. Although CEUS had a higher sensitivity (87.10%), the difference of the diagnostic accuracy was not statistically significant (P=0.406, **Table 5**). In addition, CT and CEUS had no significant difference in benign and malignant polyps (P=0.362, **Table 6**).

**Table 5:** Comparison of the diagnostic accuracy of CEUS and CT in neoplastic and non-neoplastic polypoid lesions.

	CEUS		CT	
	Neoplastic (45)	Non-neoplastic (44)	Neoplastic (39)	Non-neoplastic (50)
Pathologic diagnosis				
Neoplastic (n=31)	27	4	26	5
Non-neoplastic (n=58)	18	40	13	45
Sensitivity	87.10% (27/31)		83.87% (26/31)	
Specificity	68.97% (40/58)		77.59% (45/58)	
PPV	60.00% (27/45)		66.67% (26/39)	
NPV	90.91% (40/44)		90.00% (45/50)	
P	0.406			

PPV, Positive predictive value.

NPV, Negative predictive value.

**Table 6:** Comparison of the diagnostic accuracy of CEUS and CT in benign and malignant polypoid lesions.

	CEUS		CT	
	Benign (70)	Malignant (19)	Benign (72)	Malignant (17)
Pathologic diagnosis				
Benign (n=73)	65	8	68	5
Malignant (n=16)	5	11	4	12
Sensitivity	89.04% (65/73)		93.15% (68/73)	
Specificity	68.75% (11/16)		75.00% (12/16)	
PPV	92.86% (65/70)		94.44% (68/72)	
NPV	57.89% (11/19)		70.59% (12/17)	
P	0.362			

PPV, Positive predictive value.

NPV, Negative predictive value.

### Misdiagnosis of CT and CEUS

**Figure 5:**
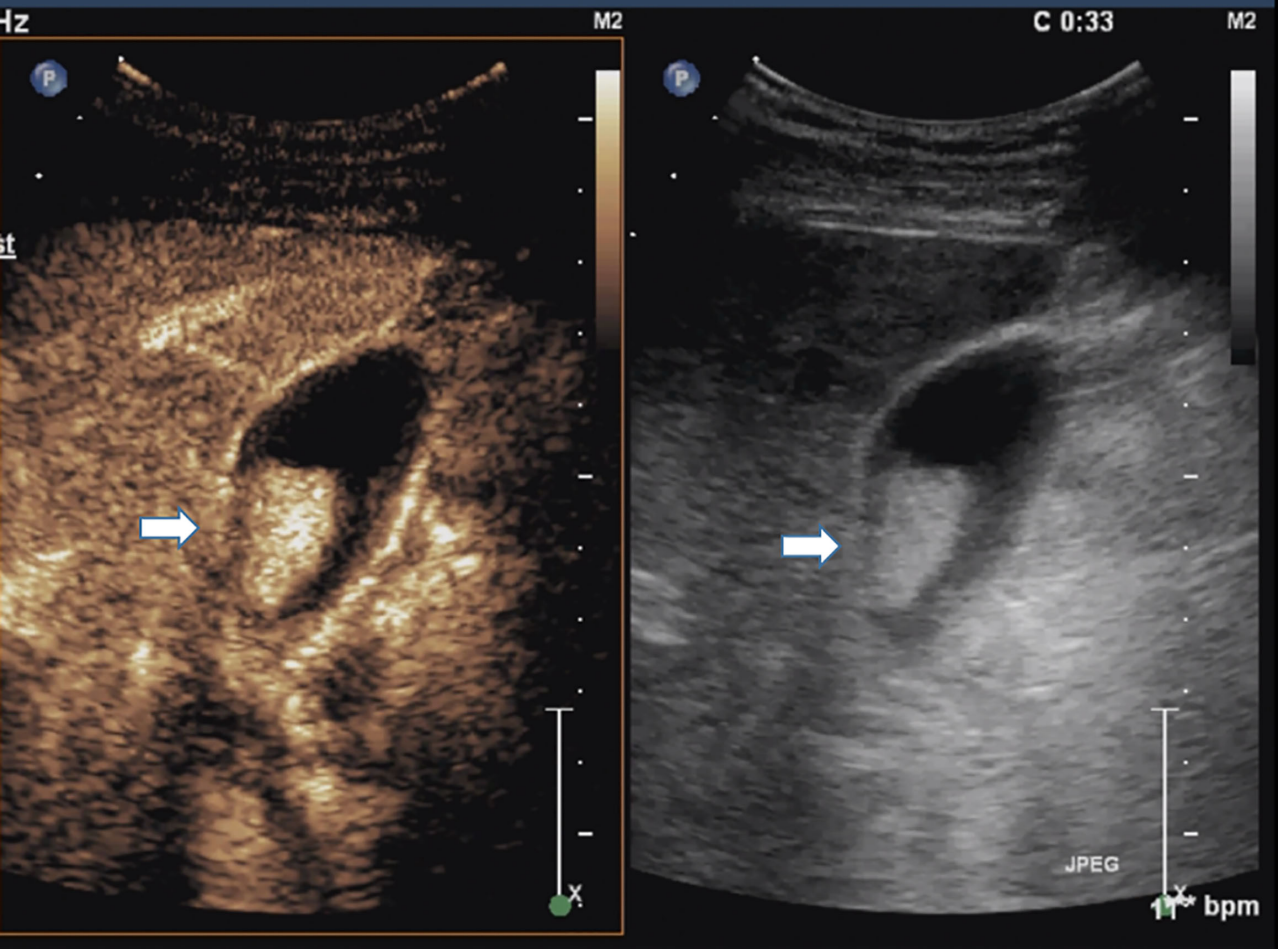
Cholesterol polyp measuring 22 mm in diameter in a 51-year-old man. Right, Conventional sonogram 2D mode (arrow). Left, Microvascular image of CEUS showing intralesional vessels with a strong and heterogeneous pattern. (arrow). So it was mistaken as gallbladder cancinoma.

**Figure 6:**
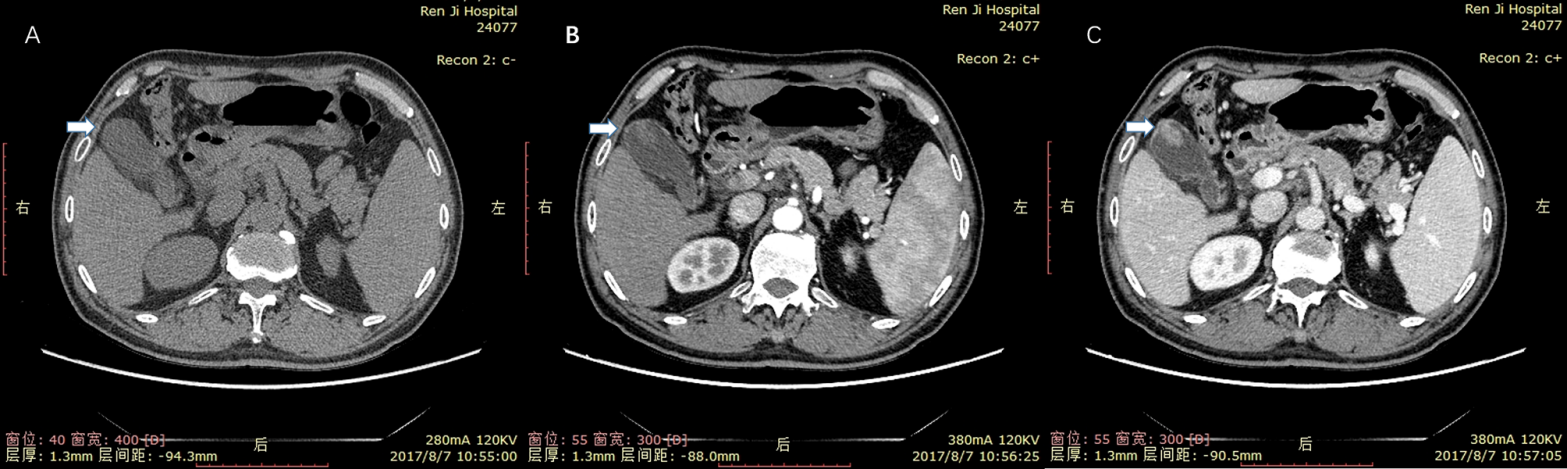
Cholesterol polyp measuring 21 mm in diameter in a 66-year-old man. **(A)** nonenhanced phase. **(B)** arterial phase. **(C)** portal venous phase. The mass could be clearly detected in the in arterial and portal venous phase. It did not have delayed enhancement characteristics of non-neoplastic polyps. So it was mistaken as gallbladder adenoma.

There were several typical misdiagnoses in the study. Misdiagnosis suggested that our lack of experience and the limitations of CT and CEUS in identifying polyps. A case of gallbladder cholesterol polyp showed blood flow on conventional sonography and a marked and homogeneous enhancement pattern on CEUS (**Figure 5**). So it was mistaken as gallbladder carcinoma. Another case of gallbladder cholesterol polyp could be clearly detected in CT arterial phase (**Figure 6**). It lacked the delayed enhancement of non-neoplastic polyps. So it was mistaken as gallbladder adenoma. The common feature of the two cases was that the cholesterol polyp is about 2 cm in diameter.

## Discussion

Asymptomatic gallbladder polyps are not uncommon, with a prevalence as high as 5% in adults. Since non-neoplastic and neoplastic polyps have different treatment strategies, it is important to obtain a preoperative clinical diagnosis. Conventional ultrasonography is used for assessment of gallbladder polyps due to its good spatial resolution, cost-effectiveness, and lack of ionizing radiation (11). However, some limitations of ultrasonography arise when characterizing gallbladder polyps. The use of contrast-specific ultrasound techniques and microbubble contrast agents in ultrasound of the liver and kidney is already well established (12). Microbubble contrast is safe and has a low risk of adverse effects, compared with CT or MRI contrast (13, 14). CEUS is good for renal impairment patient. There were no adverse reactions in 89 patients in this study. Within 2 minutes after injection, 80% of the sulfur hexafluoride microbubble should be eliminated through respiration. Fifteen minutes after injection, almost all of the gas should be eliminated. As reported in the published literature (15, 16), the incidence of severe adverse reactions that occur after the use of sulfur hexafluoride microbubbles is less than 0.01%, which is very low and shows good clinical safety

For the assessment of gallbladder polyps, CEUS is a young tool and there is relatively little literature. Nevertheless, CEUS can provide real-time, reproducible, multiplanar imaging and offers the ability to assess the vascularity of the targets with a greater sensitivity than color Doppler imaging. Previous investigations support that CEUS may be suitable for the diagnosis of gallbladder polyps. Xie et al. proved that CEUS is useful for differential diagnosis of malignant and benign gallbladder diseases with a sensitivity of 84.8% (10). Sun et al. showed that an enhancement pattern displaying branched, tortuous, or linear intralesional vessels on CEUS images was a strong differentiating factor between benign and malignant polyps (17). Fei et al. showed that the lesion size, echogenicity, and vascularity of lesions displayed on CEUS were useful for distinguishing adenoma from cholesterol polyps (18). Our previous study also supported that CEUS is a useful imaging technique and an adjunct to ultrasonography for the differential diagnosis of gallbladder polyps (19).

However, since the general acceptance of CEUS for assessing gallbladder diseases in 2007, no unified evaluation criteria have been proposed (20). Although there were significant differences between two groups in perfusion defect and microvascular architecture, image analysis is difficult and controversial even for experienced technicians in some cases. In the present study, the two technicians reached different diagnoses for 22 of the 89 cases. These disagreements occurred most often for atypical cases. This finding supports that there is still much room to improve this “young” technique.

Limited by its low detection rate, CT has long fallen out of favor. One study reported that the diagnostic accuracy of CT for distinguishing between benign and malignant polyps was only 44.4% (8). However, our study found that non-neoplastic polyps exhibit a “delayed enhancement” effect in CT scans. It is helpful to distinguish between neoplastic and non-neoplastic polyps. Several studies have found that if a lesion is pedunculated and is not visualized on nonenhanced phase, it tends to be a non-neoplastic polyp; by contrast, if a lesion is sessile and can be visualized on nonenhanced phase, it tends to be a neoplastic polyp (21, 22). In addition, because only patients with gallbladder polyps with diameters > 10 mm were included, all polyps could be displayed on CT images with a 5 mm slice thickness. This choice may have decreased interference of the low detection rate of CT.

Why are non-neoplastic polyps not displayed in the nonenhanced phase? We hypothesize that this may be due to the similar composition and x-ray permeability of non-neoplastic polyps and the surrounding bile. We further hypothesize that a relationship between bile composition and pathological type may be found by analyzing bile composition and polyp bile ratio (PBR). We aim to explore this possibility in future studies.

Diffusion-weighted MRI is another potential diagnostic tool for assessing gallbladder polyps. Studies report a sensitivity from 69–79% (23), although the sample sizes in published studies are extremely limited. Thus, the clinical value of MRI, CT, and CEUS for diagnosing gallbladder polyps warrants further comparative research.

Unlike the classification of benign and malignant polyps performed in many studies, our study focused on the differential diagnosis of non-neoplastic and neoplastic polyps to determine whether laparoscopic cholecystectomy is needed. An adenoma is a benign tumor that, due to its tendency to become malignant, requires surgery. Thus, we aimed to differentiate non-neoplastic polyps from neoplastic polyps to better guide treatment. Our study showed that CEUS and CT were both useful in clinic work. CEUS had a similar diagnostic efficacy as CT. Thus, we conclude that CEUS, when operated by experienced technicians, is an equally effective and safer tool for the diagnosis of gallbladder polyps relative to CT.

There are several shortcomings of this study. 1. The sample size is relatively small, and so studies with larger patient samples are needed to confirm our findings. 2. Image analysis is easily affected by the doctor’s observer effect and experience. 3. Laparoscopic cholecystectomy is advocated for all gallbladder polyps larger than 10 mm in China. But in the present study 65.2% of patients diagnosed with non-neoplastic polyps after surgery did not require resection although the polyps were larger than 10 mm. We plan to improve the diagnostic specificity of neoplastic polyps and revise the standard for “larger than 10mm” in future.

## Conclusion

CEUS is preferred over CT in the differential diagnosis of neoplastic and non-neoplastic gallbladder polyps due to its comparable diagnostic efficacy, lack of radiation dose and low risk of adverse effects.

## Data Availability Statement

The raw data supporting the conclusions of this article will be made available by the authors, without undue reservation.

## Ethics Statement

The studies involving human participants were reviewed and approved by The Ethics Committees of Renji Hospital affiliated to School of Medicine, Shanghai Jiao Tong University. The patients/participants provided their written informed consent to participate in this study. Written informed consent was obtained from the individual(s) for the publication of any potentially identifiable images or data included in this article.

## Author Contributions

FL and TC conceived the manuscript. ZY and XL designed and wrote the manuscript. QL, YZ, and LZ collected case data. TC and QL revised the manuscript. All authors contributed to the article and approved the submitted version.

## Funding

The research leading to these results has received funding from Program of Medicine and Engineering Cross Funding of Shanghai Jiao Tong University (YG2016MS47 to TC), Program of Clinical Research Innovation Cultivation Funding of Renji Hospital affiliated to School of Medicine Shanghai Jiao Tong University (PYZY16-017 to TC), Shanghai Outstanding Academic Leaders Plan (2016 to Jian Wang), High-Level Collaborative Innovation Team Incentive Program of Shanghai Municipal Education Commission (2018 to Jian Wang), and Foundation of Shanghai Municipal Health Commission (202040289).

## Conflict of Interest

The authors declare that the research was conducted in the absence of any commercial or financial relationships that could be construed as a potential conflict of interest.
